# Surface-plasmon-coupled optical force sensors based on metal–insulator–metal metamaterials with movable air gap

**DOI:** 10.1038/s41598-020-71825-x

**Published:** 2020-09-09

**Authors:** Taiyu Okatani, Shota Sekiguchi, Kazuhiro Hane, Yoshiaki Kanamori

**Affiliations:** 1 Department of Robotics, Tohoku University, Sendai, 980-8579 Japan; 2 Department of Finemechanics, Tohoku University, Sendai, 980-8579 Japan

**Keywords:** Nanoscience and technology, Optics and photonics

## Abstract

We proposed surface-plasmon-coupled optical force sensors based on metal–insulator–metal (MIM) metamaterials with a movable air gap as an insulator layer. The MIM metamaterial was composed of an air gap sandwiched by a metal nanodot array and a metal diaphragm, the resonant wavelength of which was red-shifted when the air gap was narrowed by applying a normal force. We designed and fabricated a prototype of the proposed sensor and confirmed that the MIM metamaterial could be used as a force sensor with larger sensitivity than a force sensor based on Fabry-Pérot interferometer (FPI).

## Introduction

Optical force sensors offer several advantages such as immunity to electromagnetic interference, no risk of electric shock in biomedical applications, and long-distance measurement using a single optical fiber without complicated wiring compared to electric force sensors^1^. A typical example of optical force sensors is a force sensor mounted based on Fabry-Pérot interferometer (FPI)^2–10^, which is utilized for monitoring various biosignals such as blood pressure^11^, intradiscal pressure^12^, intracranial pressure^13^, and intraocular pressure^14^. The sensitivity of an FPI-based force sensor depends on both the deflection of diaphragm per unit force and the resonant wavelength shift per unit deflection. However, the latter is constant in each resonant mode of FPI; for example, the value becomes 2 in the first resonant mode. Thus, the sensitivity of the sensor depends on only the deflection of diaphragm, which leads to a tradeoff between the high sensitivity and the maximum force capacity.

As with FPI, some photonic metamaterials also show an optical resonant response due to an electromagnetic interaction between an incident light and the subwavelength structure formed in them^15–22^. One of the photonic metamaterials is a metal–insulator–metal (MIM) metamaterial which shows a dip of the reflectance at a resonant wavelength^23,24^. This dip is described as the absorption of the light due to localized surface plasmon resonance on two metal layers sandwiching an insulator layer^25^, which leads to various applications of MIM metamaterials such as refractive index sensors^26^, color filters^27^, and absorbers^28^. According to previous researches, the resonant wavelength depends on the thickness of insulator layer and drastically shifts when the insulator layer becomes narrower^29^. This shift per unit thickness change can be larger than that of FPI; therefore, there is a possibility to break through the tradeoff of the existing optical force sensors. To our best knowledge, although several force/pressure/strain sensors with nanoplasmonic structure have been proposed so far^30–35^, there is no research on force sensors using the thickness change of the insulator layer in MIM metamaterials.

In this study, we propose a MIM-metamaterial-based force sensor which is composed of an air gap as an insulator layer sandwiched by a metal nanodot array and a metal diaphragm. The insulator layer is the movable air gap that changes with the deflection of diaphragm, which enable MIM metamaterials to be used as a force sensor. Compared to an FPI-based force sensor, the MIM-metamaterial-based force sensor shows larger resonant wavelength shift per unit thickness change of the air gap. Moreover, the resonant wavelength can be designed as the planar size of nanodot pattern in the MIM metamaterial while it can be designed as the thickness of optical cavity in FPI, which means the MIM-metamaterial-based sensor can be thinner when the same resonant wavelength is designed.

**Figure 1:**
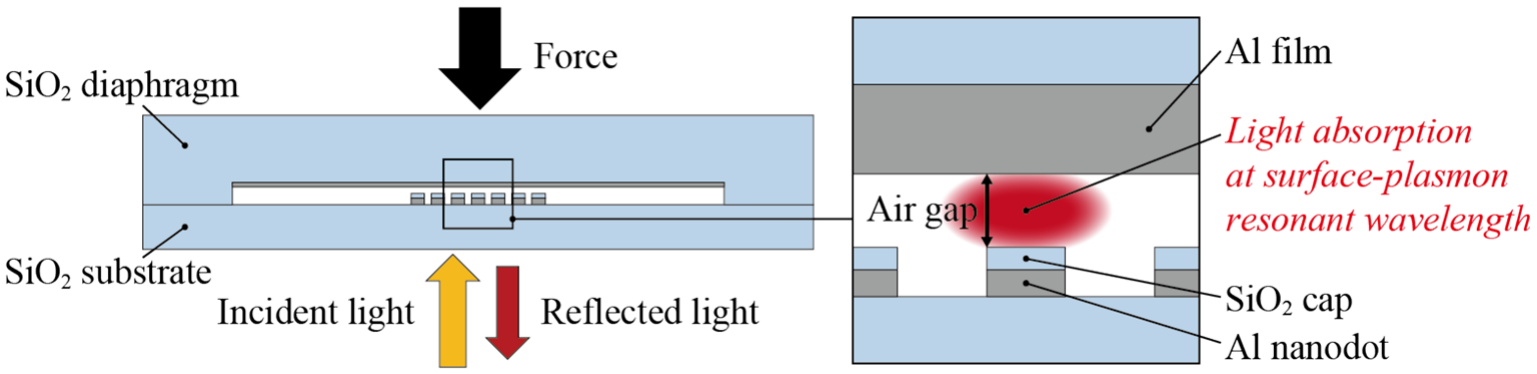
A schematic diagram of the proposed MIM-metamaterial-based force sensor.

Figure 1 shows a schematic diagram of the MIM-metamaterial-based force sensor, which is composed of an SiO_2_ diaphragm with an Al film, an air gap, an Al nanodot array, and an SiO_2_ substrate. The Al nanodot array is capped with SiO_2_ so as to prevent contact between the Al film and the Al nanodot array when the air gap is narrowed by a normal force applied on the diaphragm. Thus, the MIM metamaterial is composed of the Al film as a top metal layer, the movable air gap and the SiO_2_ cap as an insulator layer, and the Al nanodot array as a bottom metal layer. With white light incident on the bottom side of the sensor, a strong absorption peak occurs at the resonant wavelength of the MIM metamaterial and is red-shifted as the air gap is narrowed by the force. Therefore, by monitoring the resonant wavelength in the reflectance spectrum, the change of force can be detected. Note that Al is chosen as the material of MIM metamaterials because Al is found better than Au in adhesiveness to SiO_2_ and better than Ag in oxidative resistance due to the oxide film near the Al surface.

## Results

### Simulation of optical characteristics

**Figure 2:**
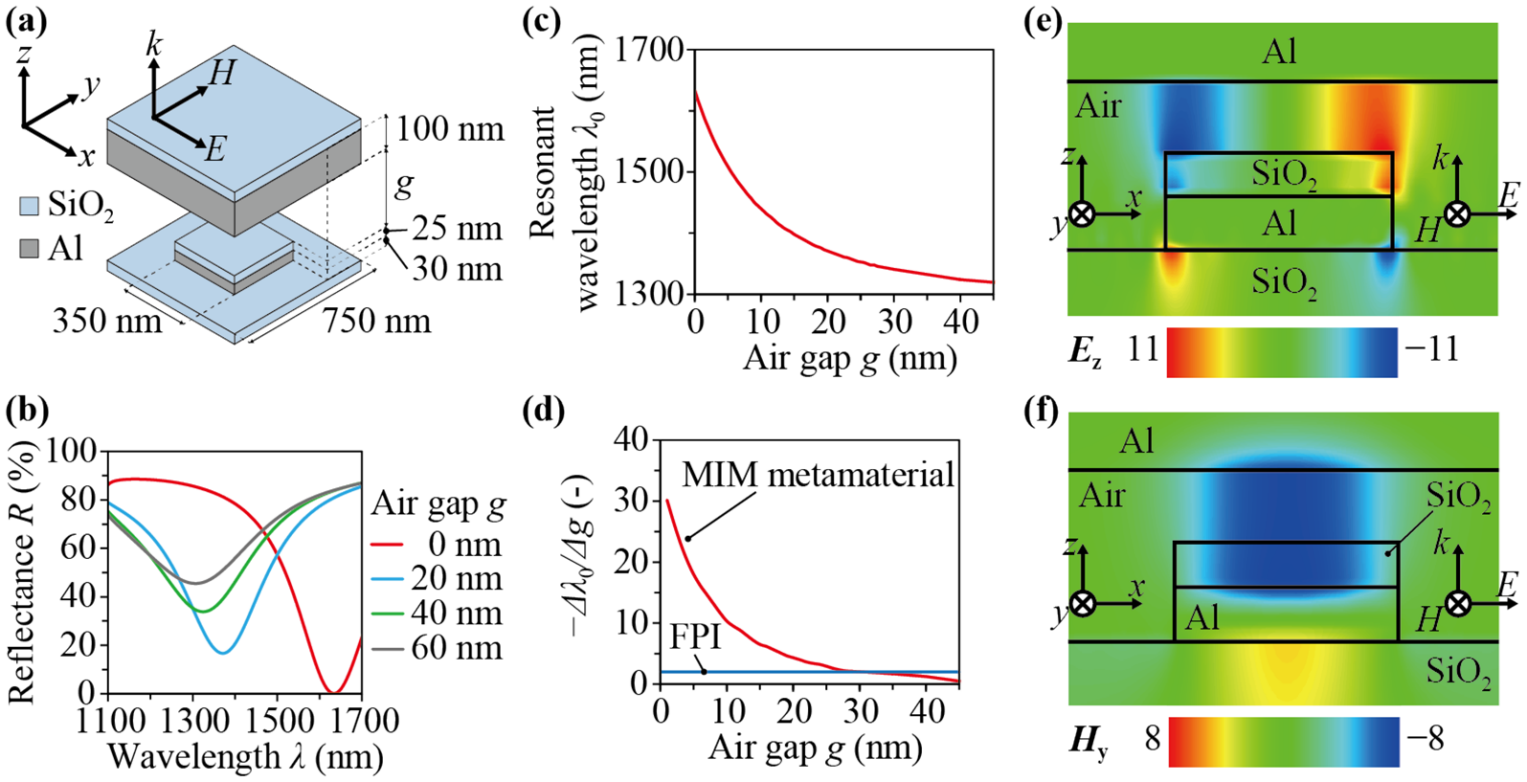
(**a**) A schematic diagram of a unit cell of the MIM metamaterial. Simulated results of (**b**) the reflectance spectra when the thickness of the air gap was 0, 20, 40, or 60 nm and (**c**) the relationship between the resonant wavelength and the thickness of air gap. (**d**) The relationships between the change rate of resonant wavelength to thickness of air gap and the thickness of air gap of the MIM metamaterial and FPI. The real parts of (**e**) the electric field and (**f**) the magnetic field in the cross section through the center of the unit cell parallel to the *x*–*z* plane at *g* = 40 nm and *λ*_0_ = 1,324 nm.

Figure 2a shows a schematic diagram of a unit cell of the nanodot array and a part of the diaphragm opposing the unit cell. The unit cell is square-shaped, 750 nm each side. The Al nanodot and SiO_2_ cap are also square-shaped at the center of the unit cell, 350 nm each side. The thicknesses of the Al nanodot and SiO_2_ cap are 30 nm and 25 nm, respectively. The Al film on the diaphragm are positioned above the unit cell with air gap being interposed therebetween, the thickness of which is represented as *g*. The thickness of the Al film is 100 nm. To investigate optical characteristic changes with respect to *g*, numerical simulations were carried out by using a commercial software (DiffractMOD, Synopsys, Inc.) based on Rigorous coupled-wave analysis (RCWA) method^36^. The linearly polarized light was irradiated on the bottom side of the MIM metamaterial vertically, and *g* was set in the range from 0 to 60 nm.

Figure 2b shows the calculated results of the reflectance spectrum. The resonant wavelengths, *λ*_0_, at *g* = 0, 20, 40, and 60 nm were 1,634, 1,370, 1,324, and 1,307 nm, respectively. It can be seen that the resonant wavelength was red-shifted, and the minimum peak value of the reflectance decreased as the air gap became narrower. Figure 2c shows the relationship between the resonant wavelength and the thickness of air gap. This relationship was not linear, and the change rate of resonant wavelength to thickness of air gap became larger as the air gap became narrower. Figure 2d shows the relationships between the change rate of resonant wavelength to thickness of air gap, − Δ*λ*_0_/Δ*g* (a minus sign is attached because *λ*_0_ decreases as *g* increases), and the thickness of air gap of the MIM metamaterial and FPI. In the case of FPI, the resonant wavelength is equal to 2 times the thickness of air gap in the first resonant mode; therefore, the change rate of resonant wavelength to thickness of air gap is a constant value, 2. In the case of the MIM metamaterial, the change rate of resonant wavelength to thickness of air gap was larger than 2 in the range of the thickness of air gap from 0 to 30 nm.

Since the change of the force on the sensor, Δ*F*, is detected as the resonant wavelength shift, Δ*λ*_0_, the sensitivity of the sensor is represented as the change rate of resonant wavelength to force, Δ*λ*_0_/Δ*F*. Furthermore, it is also represented as the product of the change rate of resonant wavelength to thickness of air gap, Δ*λ*_0_/Δ*g*, and the change rate of thickness of air gap to force, Δ*g*/Δ*F*. Because the latter depends on the mechanical properties of the diaphragm, the difference of the sensitivities between the MIM-metamaterial-based force sensor and FPI-based force sensors is determined by Δ*λ*_0_/Δ*g* if the same diaphragm is used. From the above results, it can be seen that the sensitivity of the MIM-metamaterial-based sensor is superior to that of an FPI-based force sensor in the range of the thickness of air gap from 0 to 30 nm.

Figures 2e,f show the real parts of the electric field and the magnetic field in the cross section through the center of the unit cell parallel to the *x*–*z* plane at *g* = 40 nm and *λ*_0_ = 1,324 nm, which is the resonant wavelength at *g* = 40 nm, respectively. Enhancements of the electric and magnetic fields were confirmed inside the insulator layer made of the air gap and the SiO_2_ cap, which indicates that the confinement of electro-magnetic energy in the MIM metamaterial leads to the dissipation of the incident light at the resonant wavelength corresponding to the thickness of air gap as mentioned in the previous research^25^.

### Sensor fabrication

**Figure 3:**
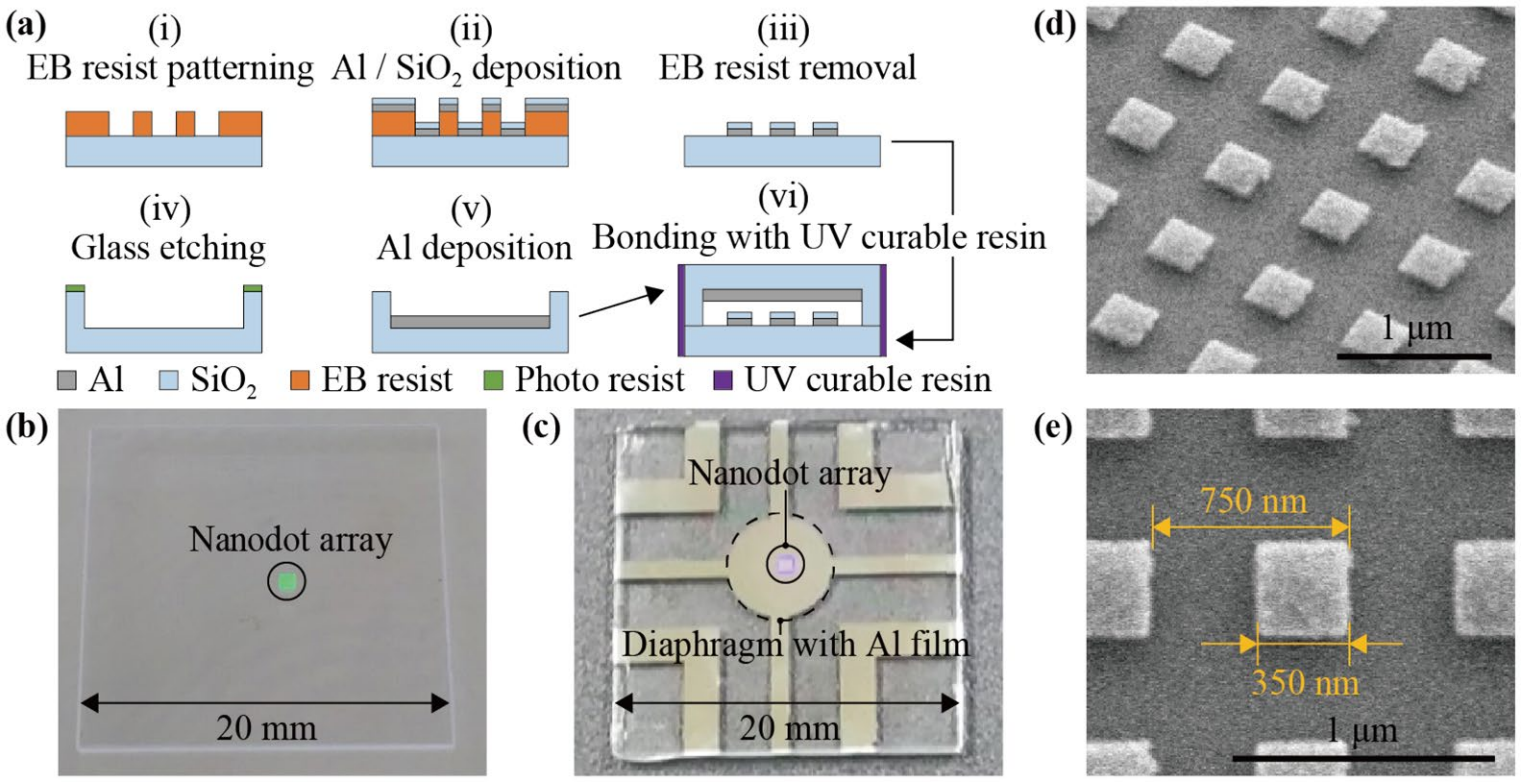
(**a**) Fabrication processes of the MIM-metamaterial-based force sensor. Photographs of (**b**) the SiO_2_ substrate with the nanodot array and (**c**) the fabricated sensor after bonding the SiO_2_ substrate and the SiO_2_ diaphragm. SEM images of (**d**) an overview and (**e**) a unit cell of the nanodot array.

Figure 3a shows schematic diagrams of the fabrication processes. First, an SiO_2_ substrate with a nanodot array of the sensor was fabricated using a 0.5-mm-thick glass substrate as a starting substrate. Electron-beam (EB) resist was spin-coated on the glass substrate and patterned by EB lithography. Then, Al and SiO_2_ were deposited with thicknesses of 30 and 25 nm by EB vapor deposition, respectively. The nanodot array was fabricated by removing the EB resist and Al/SiO_2_ layer on it by lift-off process. Next, an SiO_2_ diaphragm with an Al film was fabricated using a 1-mm-thick glass substrate as a starting substrate. The diameter of the diaphragm was 6 mm. Patterning photo resist as a mask, the glass substrate was etched by a 200-nm-depth by ion-beam milling. Then, the Al film was fabricated by depositing Al by a 100-nm-thickness by EB vapor deposition and removing unnecessary photoresist and Al layers. Finally, the SiO_2_ substrate with the nanodot array and the SiO_2_ diaphragm with the Al film were bonded by curing UV curable resin around the contact region between them. With the above fabrication processes, the initial thickness of air gap *g*_0_ should be 45 nm. However, we observed interference fringes on the contact region between the SiO_2_ substrate and the SiO_2_ diaphragm, which indicated some gap existed in the contact region and the initial thickness of air gap was larger than 45 nm. One of the possible causes of the gap is the presence of particles left on the contact region.

Figure 3b shows a photograph of the SiO_2_ substrate with the nanodot array. The nanodot array was fabricated in a 1 mm square near the center of the SiO_2_ substrate. Figure 3c shows a photograph of the fabricated sensor after bonding the SiO_2_ substrate and the SiO_2_ diaphragm. Figure 3d,e shows scanning electron microscopy (SEM) images of an overview and a unit cell of the nanodot array. From the SEM observation, it was confirmed that the nanodot array was fabricated with sufficient accuracy to match the design value.

### Evaluation of sensitivity

**Figure 4:**
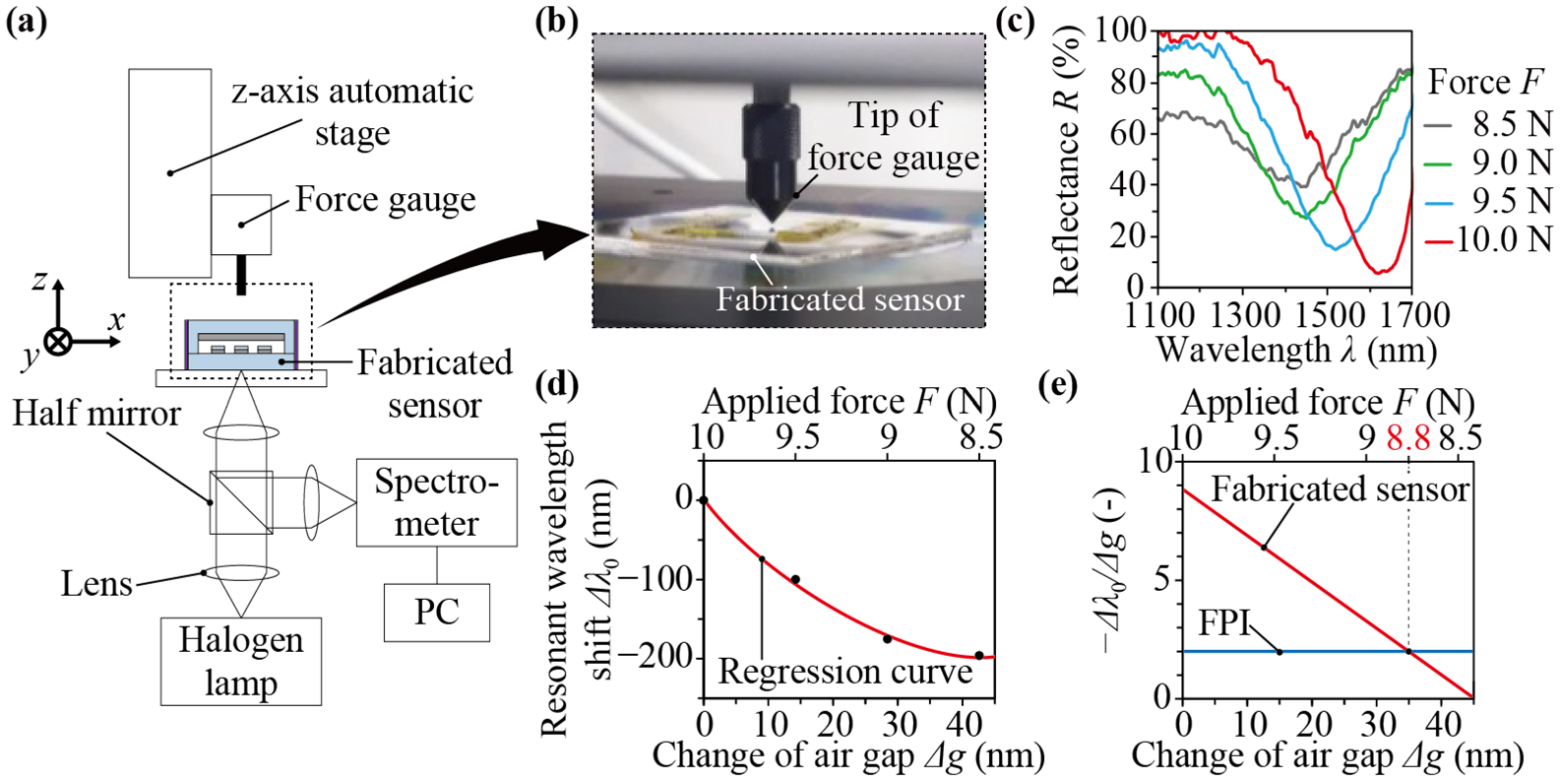
(**a**) Experimental setup for measurement of optical characteristic change of the fabricated MIM metamaterial while a normal force is applied. (**b**) A photograph of the tip of the force gauge pushing the diaphragm of the fabricated sensor. (**c**) Measurement results of the reflectance spectra. The relationships (**d**) between the resonant wavelength shift and the change of air gap and (**e**) between the change rate of resonant wavelength to thickness of air gap and the change of air gap.

Change of the optical characteristics of the fabricated sensor was measured by applying a normal force on the top side of the sensor. Figure 4a shows the experimental setup, which was composed of a *z*-axis automatic stage, a force gauge, the fabricated sensor, a halogen lamp, a half mirror, lenses, a spectrometer and PC for data acquisition. A normal force was applied by pushing the sensor with the tip of the force gauge which was moved vertically by the z-axis automatic stage as shown in Fig. 4b. White light emitted by the halogen lamp was incident on the bottom side of the sensor through the half mirror and lenses, was reflected on the MIM metamaterial, and entered in the spectrometer. Figure 4c shows the measured reflectance spectra when the force, *F*, was 8.5, 9.0, 9.5, and 10.0 N. As the force increased, the resonant wavelength was red-shifted and the reflectance at the resonant wavelength decreased, which corresponded to the simulated results shown in Fig. 2b. The resonant wavelengths at *F* = 8.5, 9.0, 9.5, and 10.0 N were 1,425, 1,446, 1,521, and 1,621 nm, respectively. Note that the force unit N is used instead of the pressure unit Pa because it is difficult to apply uniform pressure to the diaphragm with the experimental setup.

To evaluate the change rate of resonant wavelength to thickness of air gap of fabricated sensor, we estimated the change of air gap, Δ*g*, corresponding to the applied force. We used the relationship between the deflection change of diaphragm, Δ*w* =  − Δ*g*, and the force change, Δ*F*, which depends on the mechanical properties of the diaphragm. According to the theory of plates and shells^37^, the deflection change at the center of the diaphragm where point load is applied is represented as Δ*w* = 3*a*^2^(1 − *ν*^2^)Δ*F*/(4π*Eh*^3^); here, *a* is the radius, *ν* is the Poisson’s ratio, *E* is the Young’s modulus, and *h* is the thickness of the diaphragm. By substituting the design values (*a* = 3 mm, *h* = 1 mm) and the material properties of glass (*E* = 71.6 GPa, *ν* = 0.23) to the above equation and considering *F* = 10.0 N as a reference point, the changes of air gap, Δ*g*, at *F* = 8.5, 9.0, 9.5, and 10.0 N were estimated as 42.6, 28.4, 14.2, and 0 nm, respectively. Also, the resonant wavelength shifts, Δ*λ*_0_, at *F* = 8.5, 9.0, 9.5, and 10.0 N were − 196, − 175, − 100, and 0 nm, respectively. Figure 4d shows the relationship between the resonant wavelength shift and the estimated change of air gap of fabricated sensor, where the quadratic regression curve is plotted together. This relationship was not linear as predicted in the simulation result shown in Fig. 2c. Figure 4e shows the change rate of resonant wavelength to thickness of air gap of fabricated sensor, which was calculated by differentiating the regression curve shown in Fig. 4d. The change rate of fabricated sensor was larger than that of FPI in the range of the applied force from 8.8 to 10.0 N. This result shows the MIM-metamaterial-based force sensor has larger sensitivity than an FPI-based force sensor in this range if the same diaphragm is used. Because the range where the sensitivity is higher can be controlled by changing the stiffness of the diaphragm and the initial thickness of air gap, the conventional tradeoff between the sensitivity and the maximum force capacity of FPI-based force sensors can be broken through in the desired range of use.

## Discussion

We proposed the MIM-metamaterial-based force sensor with a movable insulator layer made of an air gap. The MIM metamaterial is composed of the Al film on the SiO_2_ diaphragm as a top metal layer, the Al nanodot array on the SiO_2_ substrate as a bottom metal layer, and the air gap and the SiO_2_ cap on the nanodot as an insulator layer. Enhancements of the electric and magnetic fields inside the MIM metamaterial were confirmed by RCWA simulation. A prototype of the MIM-metamaterial-based force sensor was designed and fabricated. The fabricated sensor shows the resonant wavelength shift along with the change of the force applied on the diaphragm. By estimating the change of air gap corresponding to the force, the relationship between the change rate of resonant wavelength to thickness of air gap and the change of air gap were evaluated. The result shows the MIM-metamaterial-based force sensor has larger sensitivity than an FPI-based force sensor in the range of the force from 8.8 to 10 N if the same diaphragm is used, which is promising because the tradeoff between the sensitivity and the maximum force capacity inherent in FPI-based force sensors can be break thorough.

While the sensitivity is remarkable, one of the limitations of the proposed sensor is the difficulty in fabricating a nanoscale air gap with the same thickness as the design value, which leads to a variation of the sensitivity. In order to improve the fabrication accuracy, it is necessary to optimize the fabrication processes and conditions. Especially, the bonding process of the SiO_2_ diaphragm and the SiO_2_ substrate may affect the sensitivity heavily. The presence of substances that remain on the bonded surface will increase the initial thickness of air gap. Also, because of the stiffness of the substances themselves, the deflection of the diaphragm does not match the change of air gap, that is, Δ*w* ≠  − Δ*g*. Thus, the estimated change of air gap shown in Fig. 4d,e includes an error, which is the reason why the characteristic of the fabricated sensor shown in Fig. 4e seems to be linear unlike the simulation result shown in Fig. 2d.

Another limitation is the sensitivity change due to the position of the point load applied on the diaphragm. If the position of point load deviates from the center of the diaphragm, the relationship between the force and the change in air gap varies. The same can be said when a distributed pressure is applied. In practical use, the position of the point load must be fixed not to change the sensitivity. As a future work, sensor housings that transfers a point load to the center of the diaphragm need to be designed and combined with the proposed MIM-metamaterial-based sensor as a force-sensing element.

## Methods

### Simulation conditions

The simulations were conducted with a commercial software (DiffractMOD, Synopsys, Inc.) as follows. Both width and height of a unit cell were 750 nm. Both width and height of Al nanodot and SiO_2_ cap were 350 nm. The thickness of Al nanodot and SiO_2_ cap were 30 nm and 25 nm, respectively. Al film was placed away from the top of the SiO_2_ cap by the thickness of air gap, *g*. The calculation area was set from 50 nm below the Al nanodot to 30 nm above the air gap. The thickness of air gap, g, was set in the range from 0 to 30 nm with 1 nm step while in the range from 30 to 60 nm with 5 nm step. The materials of components were chosen from Rsoft library built in by default. In the calculation of reflective spectra, harmonics and wavelength step parameters were set to 6 and 2 nm, respectively. In the calculation of electric and magnetic fields, harmonics was set to 13.
